# Supplementary material for: Socioeconomic and Governance Factors Disentangle the Relationship between Temperature and Antimicrobial Resistance: A 10-Year Ecological Analysis of European Countries
Source: Antibiotics (Basel). 2023 Apr 19;12(4):777. doi: 10.3390/antibiotics12040777 (PMC10135271; doi:10.3390/antibiotics12040777)
Supplement: Supplementary file 1 [file antibiotics-12-00777-s001.zip › antibiotics-2296250-supplementary.pdf]

Figure S1. Trends in AMR (%) for E. coli, K. pneumoniae, and S. aureus between 2000-2019 across 30 European countries. Antibiotic classes are represented by coloured lines.

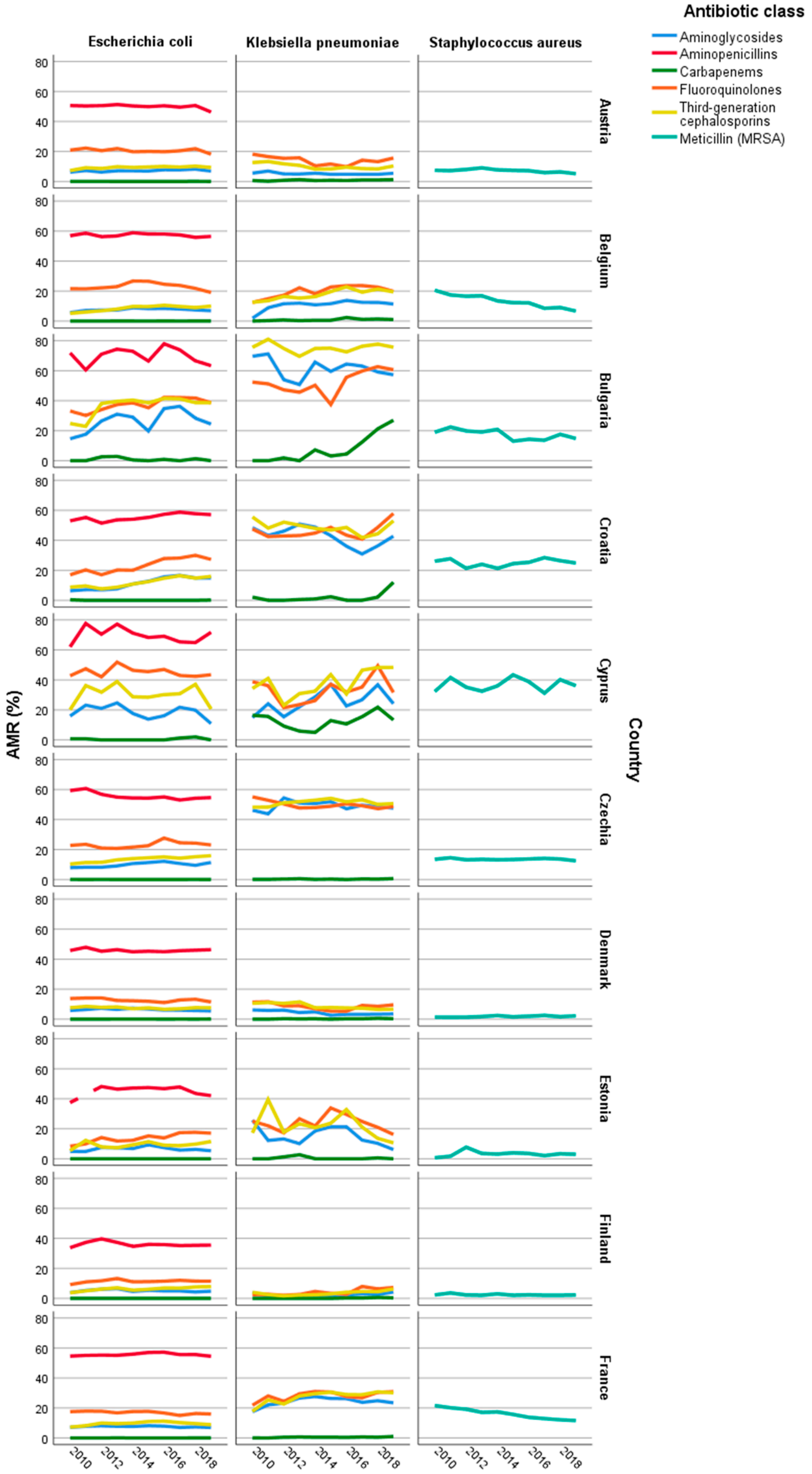

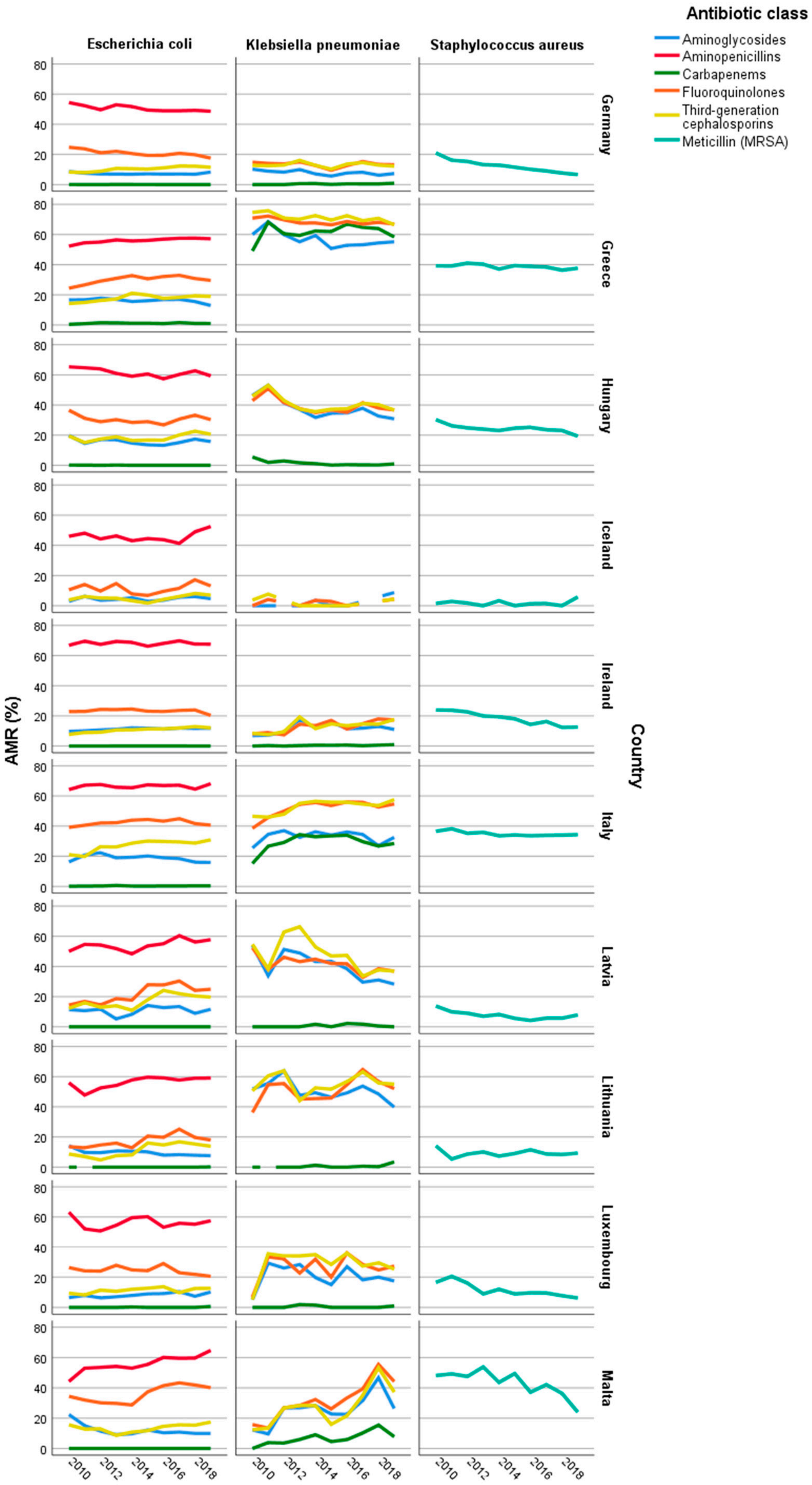

Antibiotic class
Aminoglycosides
Aminopenicillins
Carbapenems
Fluoroquinolones
Third-generation cephalosporins
Meticillin (MRSA)
Escherichia coli
Klebsiella pneumoniae
Staphylococcus aureus
Germany
Greece
Hungary
Iceland
Ireland
Italy
Latvia
Lithuania
Luxembourg
Malta
AMR (%)
Country
0
20
40
60
80
2010
2012
2014
2016
2018

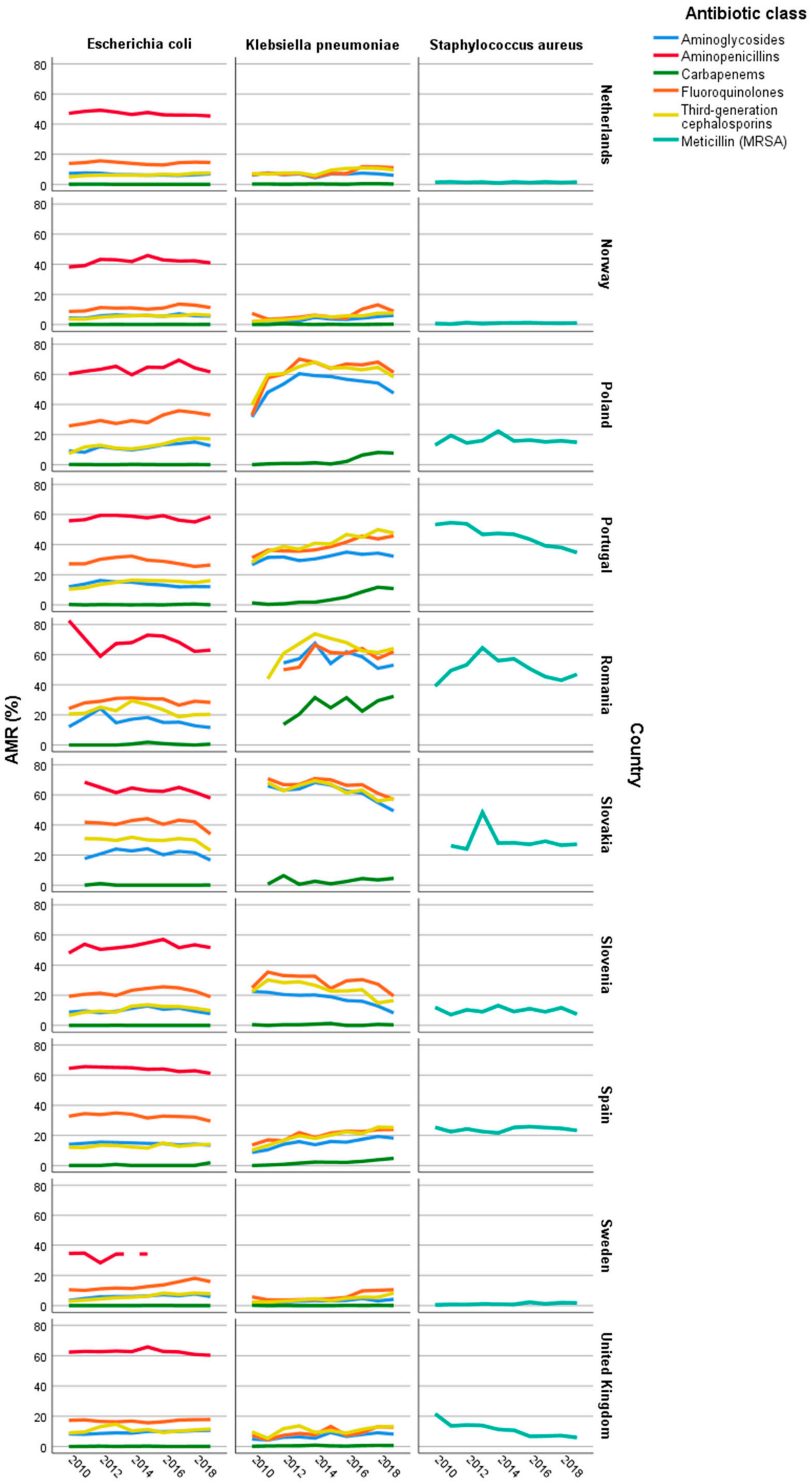
Antibiotic class
Aminoglycosides
Aminopenicillins
Carbapenems
Fluoroquinolones
Third-generation cephalosporins
Meticillin (MRSA)
Escherichia coli
Klebsiella pneumoniae
Staphylococcus aureus
Netherlands
Norway
Poland
Portugal
Romania
Slovakia
Slovenia
Spain
Sweden
United Kingdom
AMR (%)
Country
0
20
40
60
80
2010
2012
2014
2016
2018

Figure S2. Trends in annual temperature change between 2010-2019 across 30 European countries.

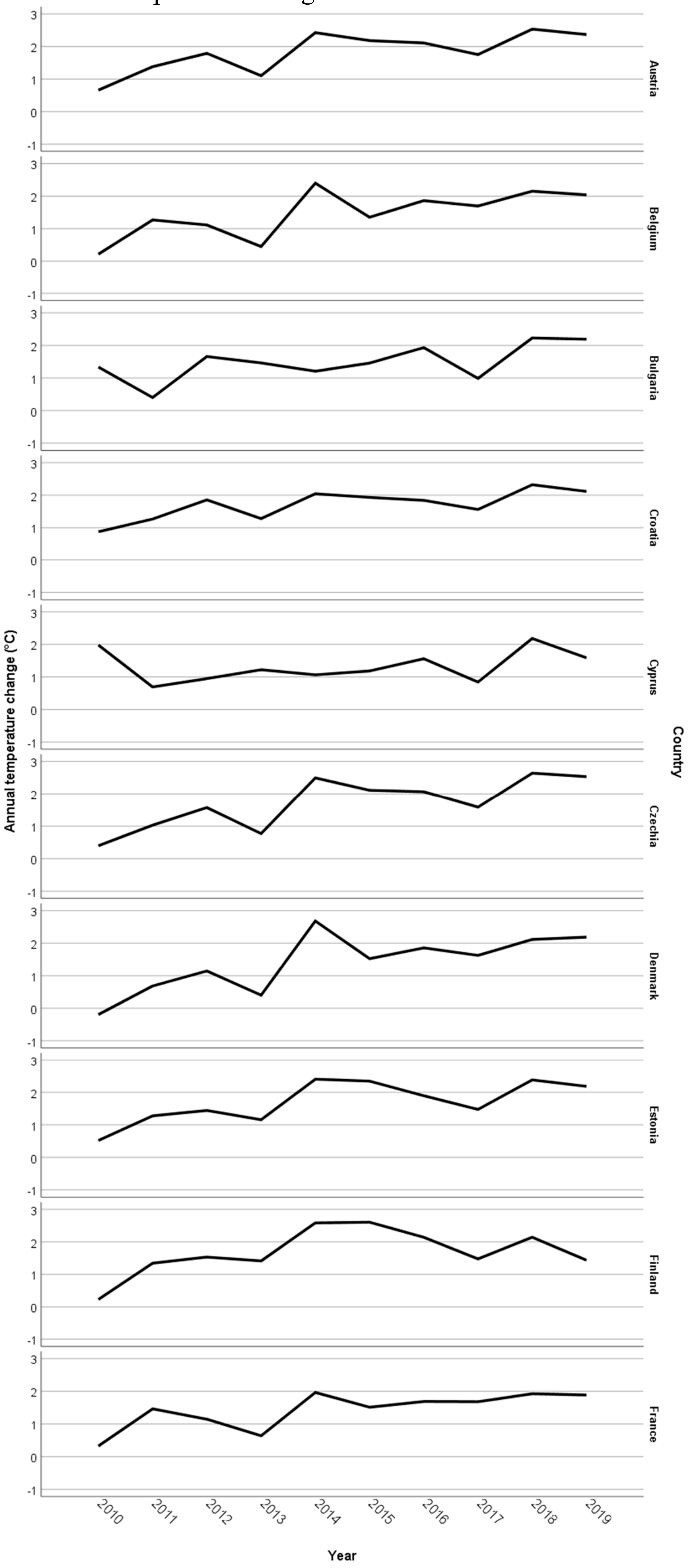

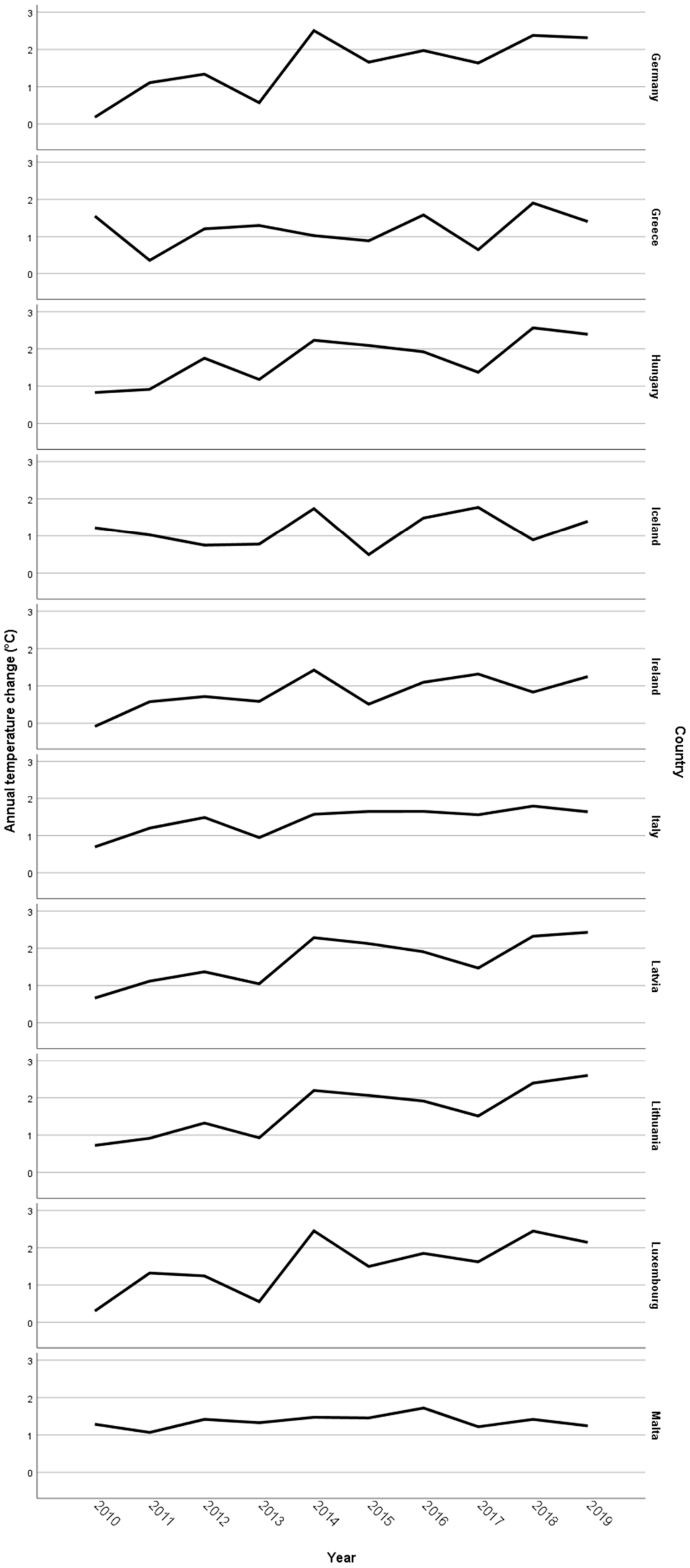
Annual temperature change (°C)
Germany
Greece
Hungary
Iceland
Ireland
Italy
Latvia
Lithuania
Luxembourg
Malta
Country
2010
2011
2012
2013
2014
2015
2016
2017
2018
2019
Year

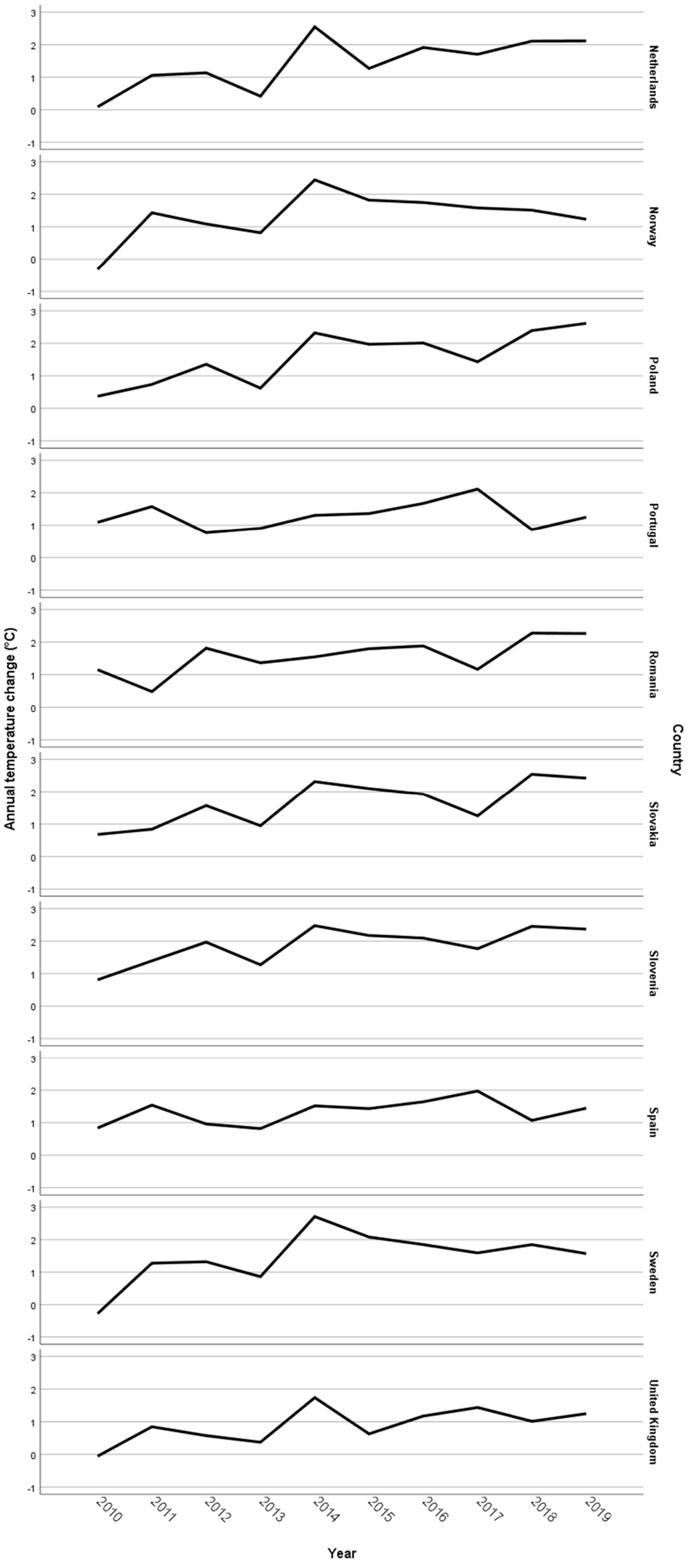

Annual temperature change (°C)
Country
Netherlands
Norway
Poland
Portugal
Romania
Slovakia
Slovenia
Spain
Sweden
United Kingdom
3
2
1
0
-1
2010
2011
2012
2013
2014
2015
2016
2017
2018
2019
Year

Figure S3. Association of annual temperature change with population density (A), GDP per capita (B), and the governance index (C). Unadjusted weighted linear trend lines are shown.

A

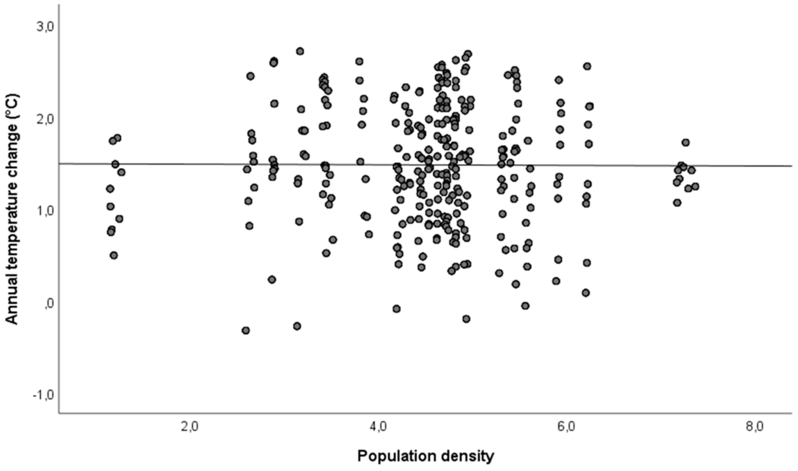

B

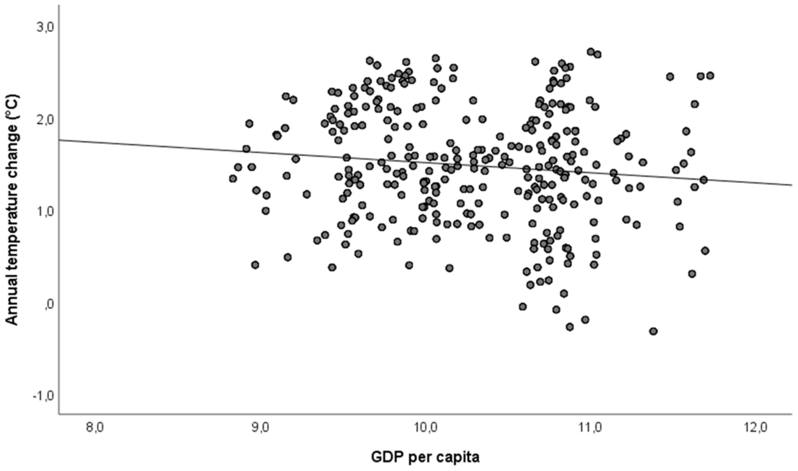

C

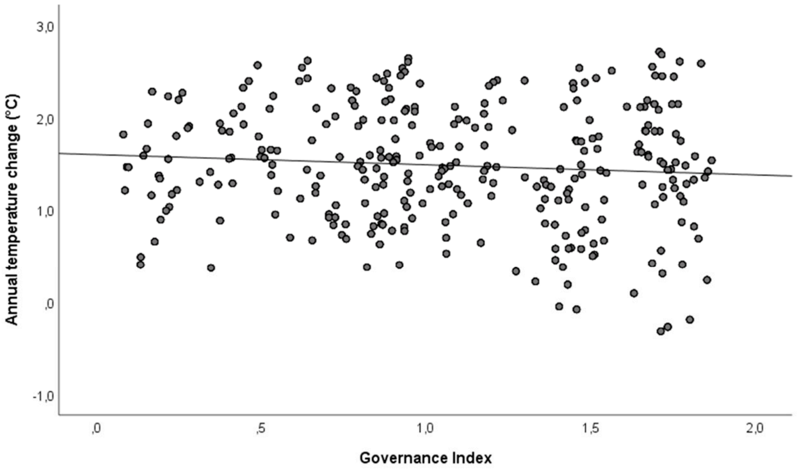

Figure S4. Trends in antibiotic consumption between 2010-2019 across 30 European countries.

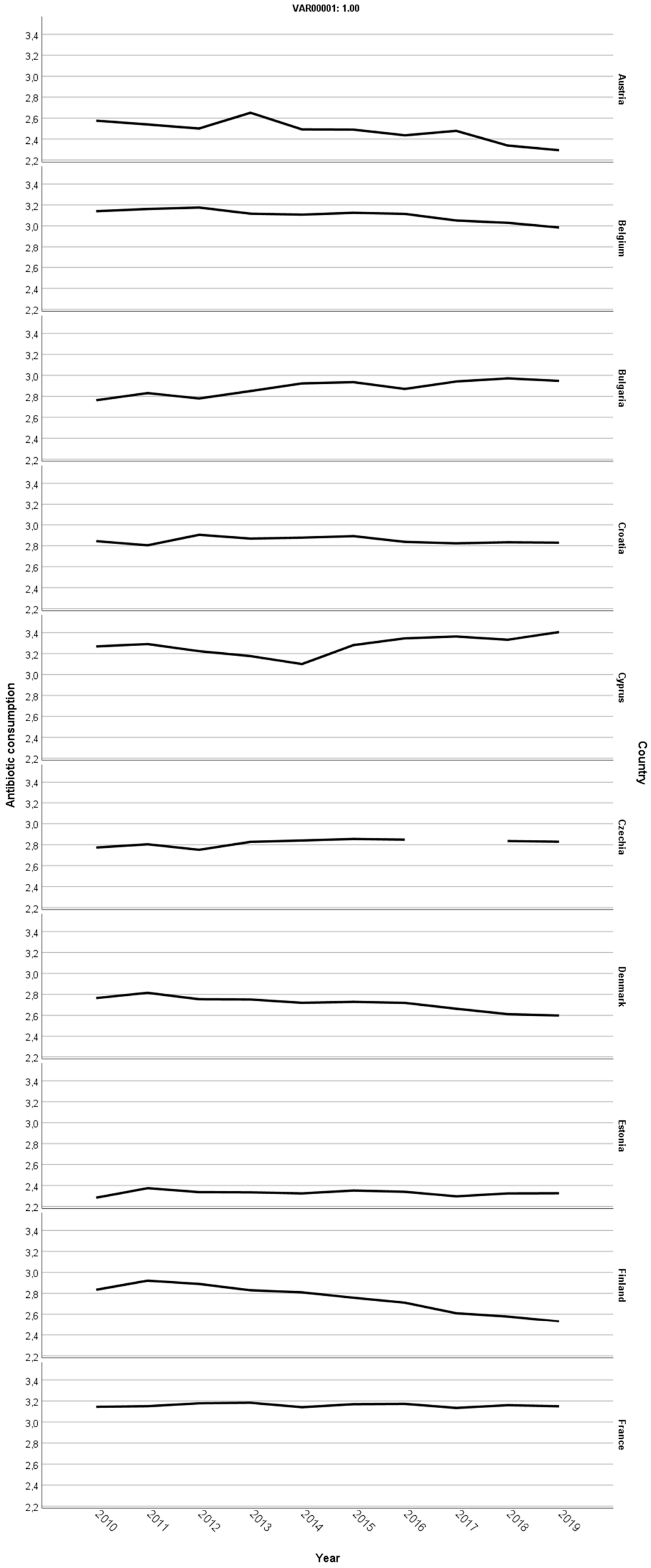

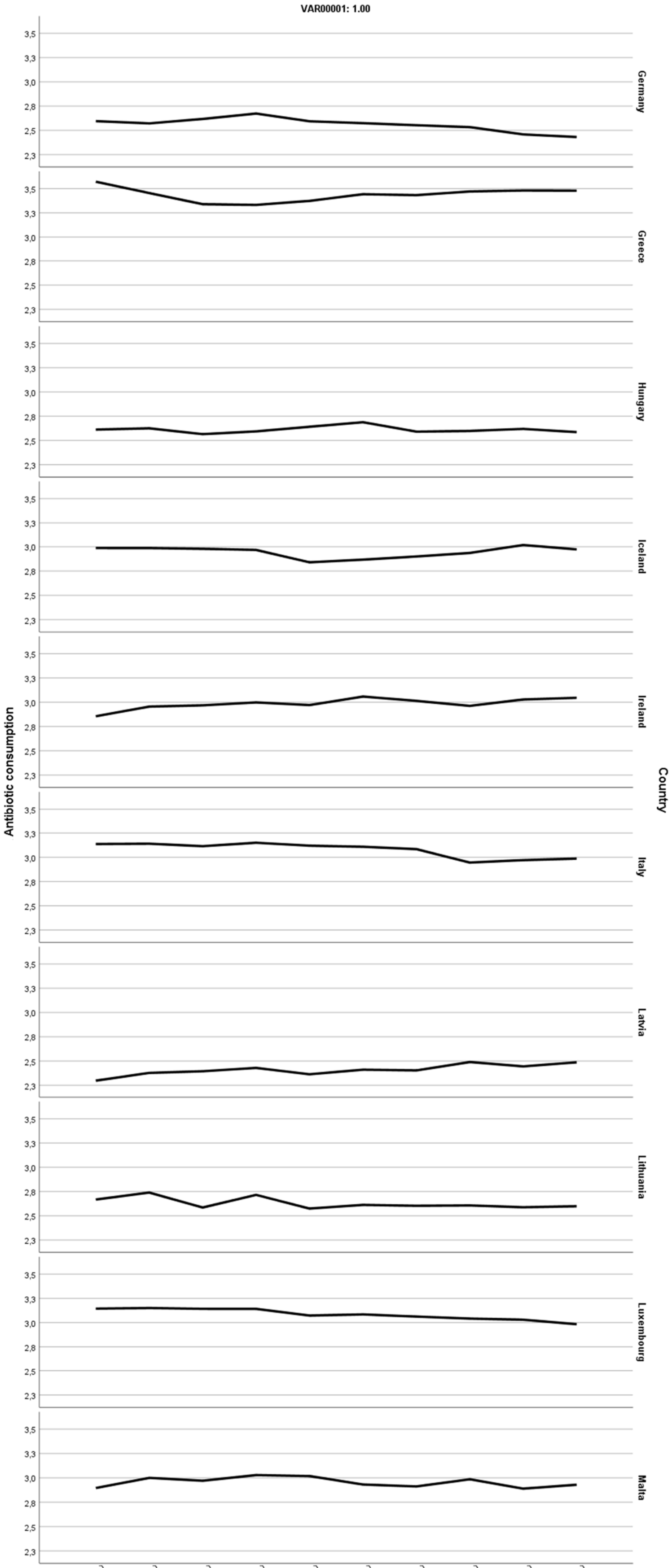
VAR00001: 1.00
Antibiotic consumption
Country
Germany
Greece
Hungary
Iceland
Ireland
Italy
Latvia
Lithuania
Luxembourg
Malta
3,5
3,3
3,0
2,8
2,5
2,3
2010
2011
2012
2013
2014
2015
2016
2017
2018
2019
Year

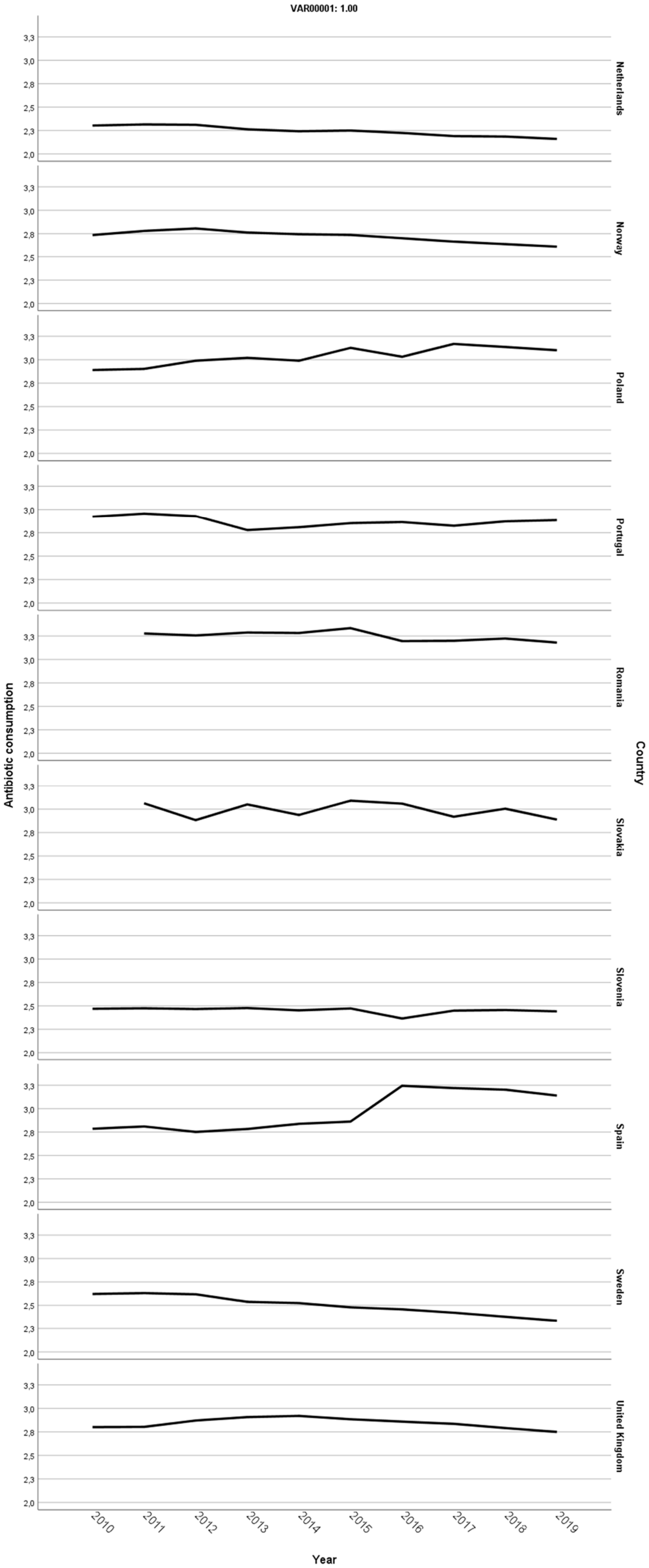

VAR00001: 1.00
Antibiotic consumption
Country
Netherlands
Norway
Poland
Portugal
Romania
Slovakia
Slovenia
Spain
Sweden
United Kingdom
3,3
3,0
2,8
2,5
2,3
2,0
2010
2011
2012
2013
2014
2015
2016
2017
2018
2019
Year

Figure S5. Trends in population density between 2010-2019 across 30 European countries.

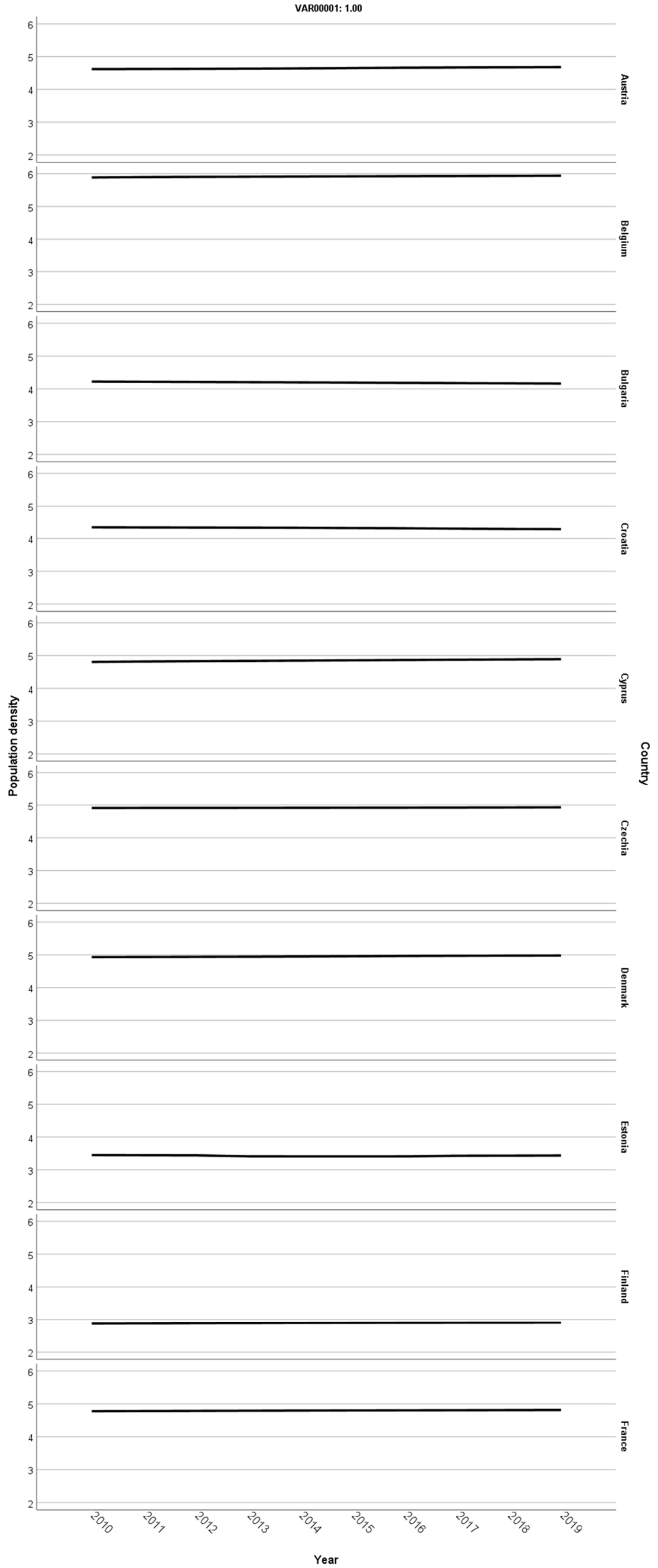

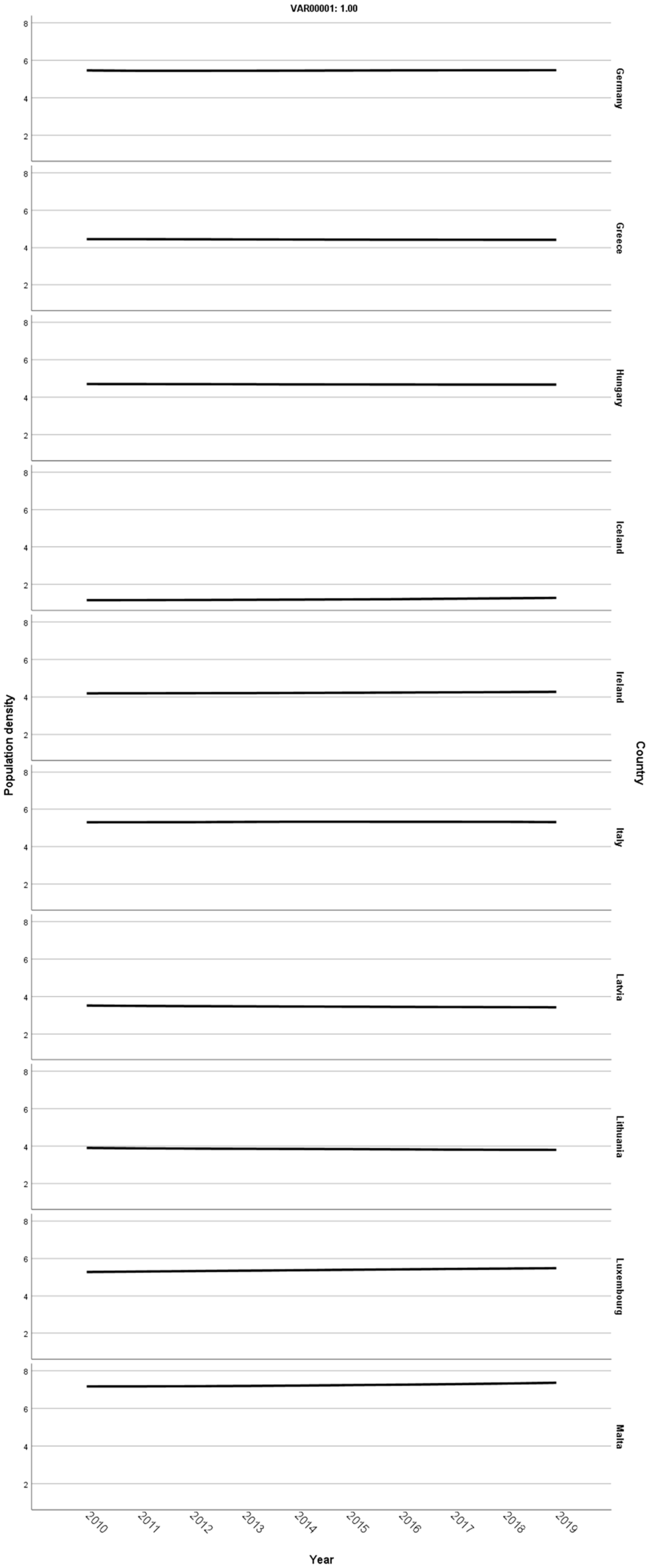
VAR00001: 1.00
Population density
Country
Germany
Greece
Hungary
Iceland
Ireland
Italy
Latvia
Lithuania
Luxembourg
Malta
Year
2010
2011
2012
2013
2014
2015
2016
2017
2018
2019

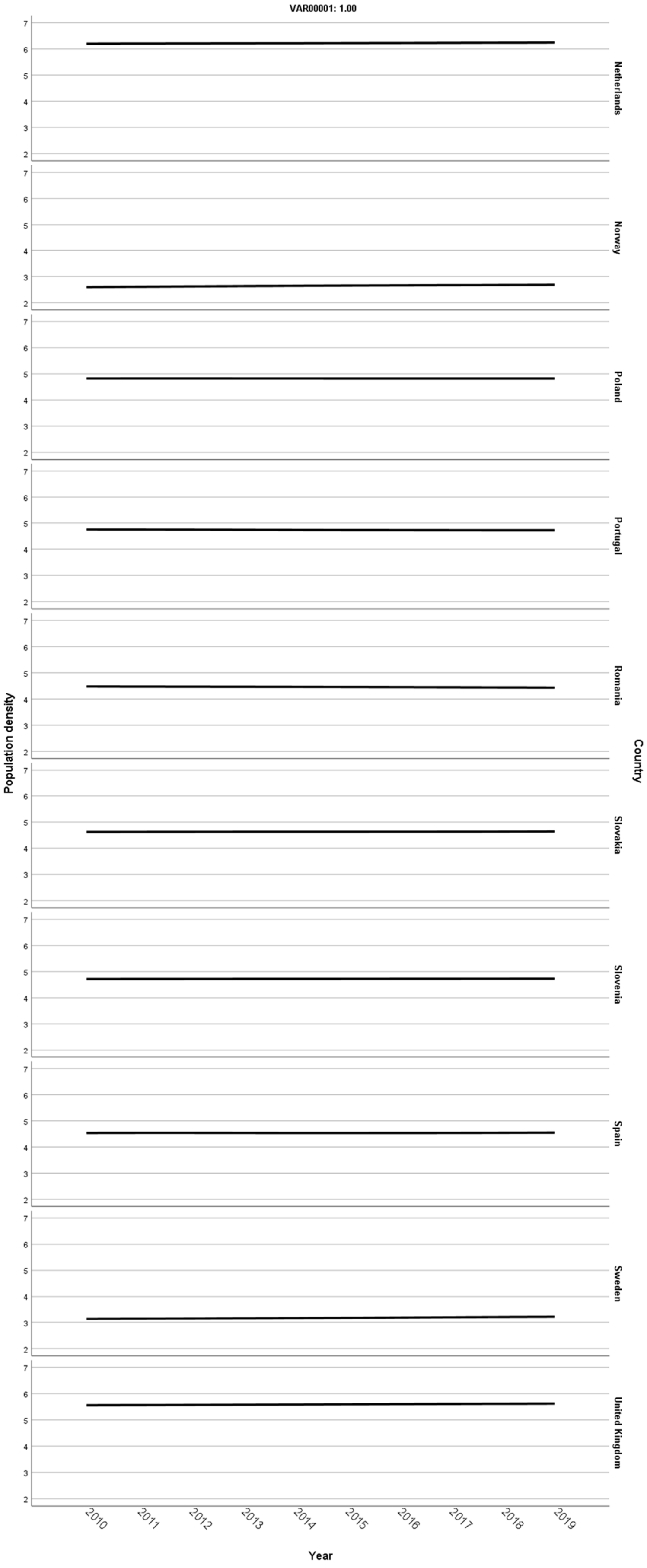

VAR00001: 1.00
Population density
Country
Netherlands
Norway
Poland
Portugal
Romania
Slovakia
Slovenia
Spain
Sweden
United Kingdom
2010
2011
2012
2013
2014
2015
2016
2017
2018
2019
Year

Figure S6. Association of antimicrobial resistance (normalized) with antibiotic consumption (A) and population density (B). Unadjusted weighted linear trend lines are shown.

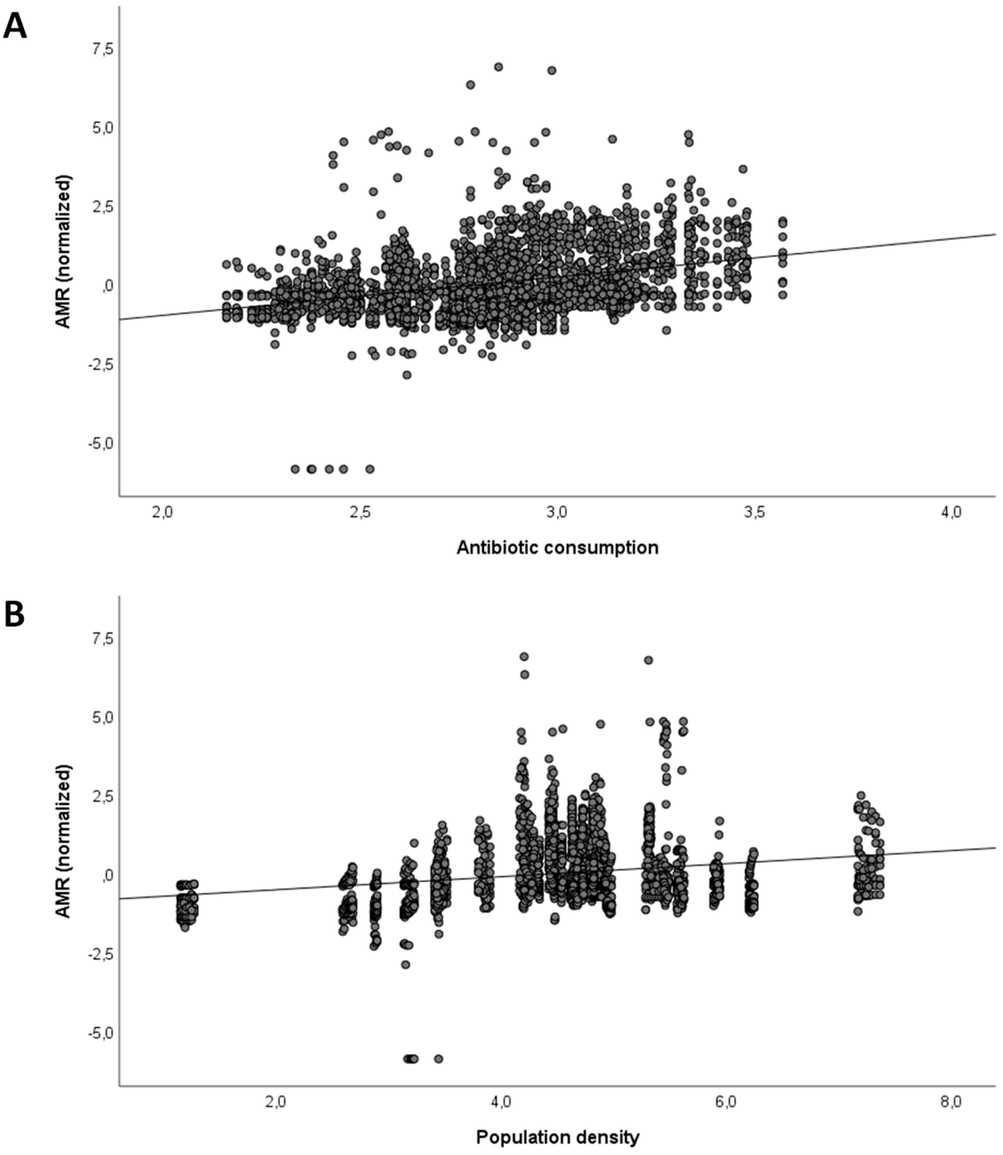

Figure S7. Trends in GDP per capita between 2010-2019 across 30 European countries.

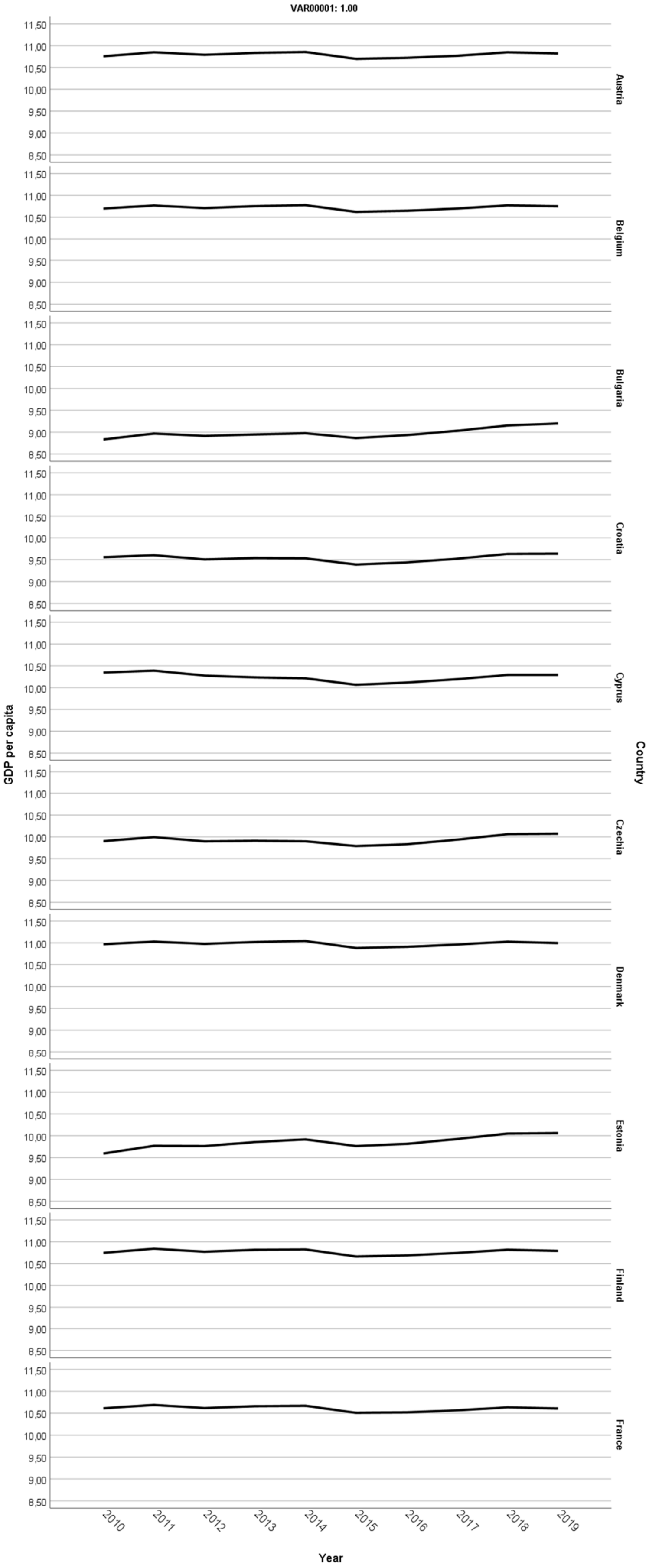

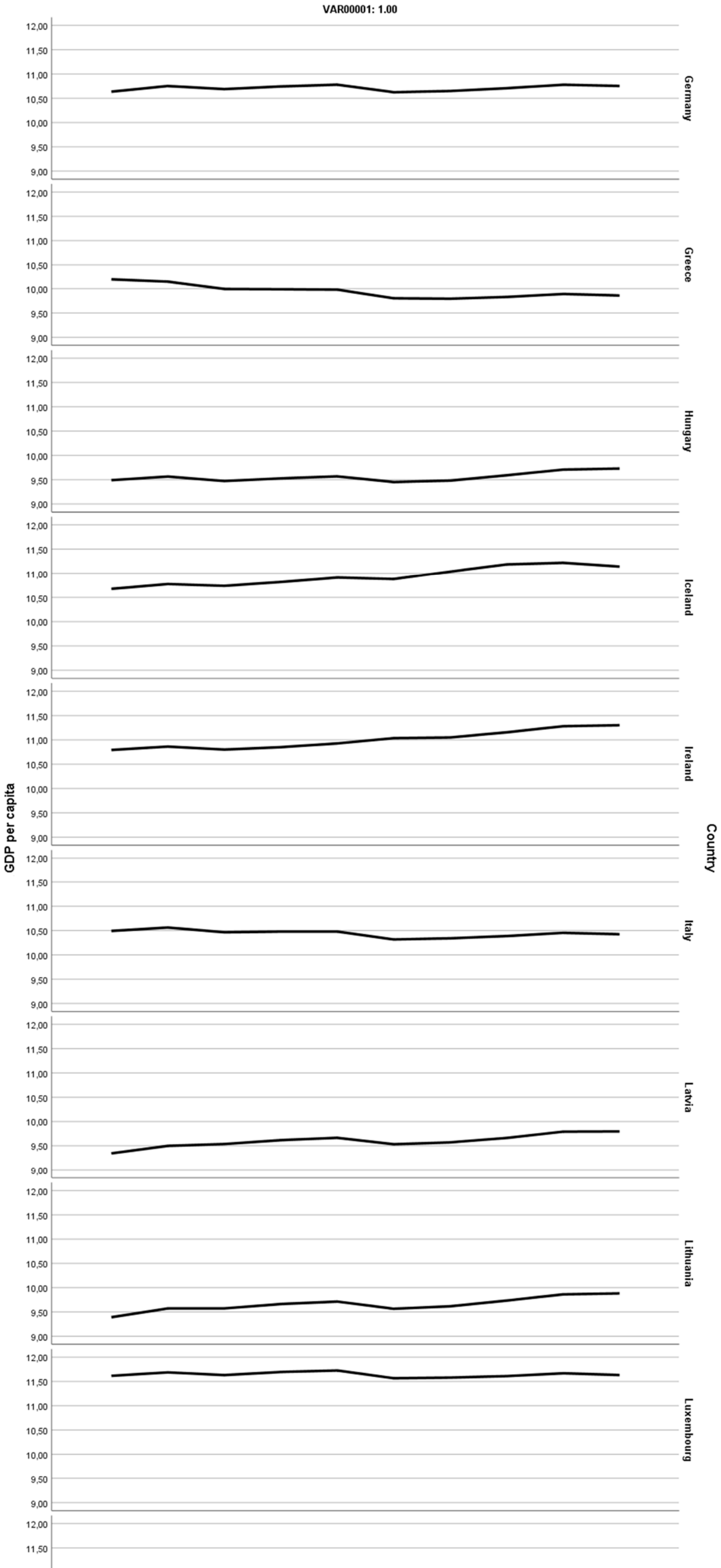
VAR00001: 1.00
GDP per capita
Country
Germany
Greece
Hungary
Iceland
Ireland
Italy
Latvia
Lithuania
Luxembourg
Malta
Year
2010
2011
2012
2013
2014
2015
2016
2017
2018
2019

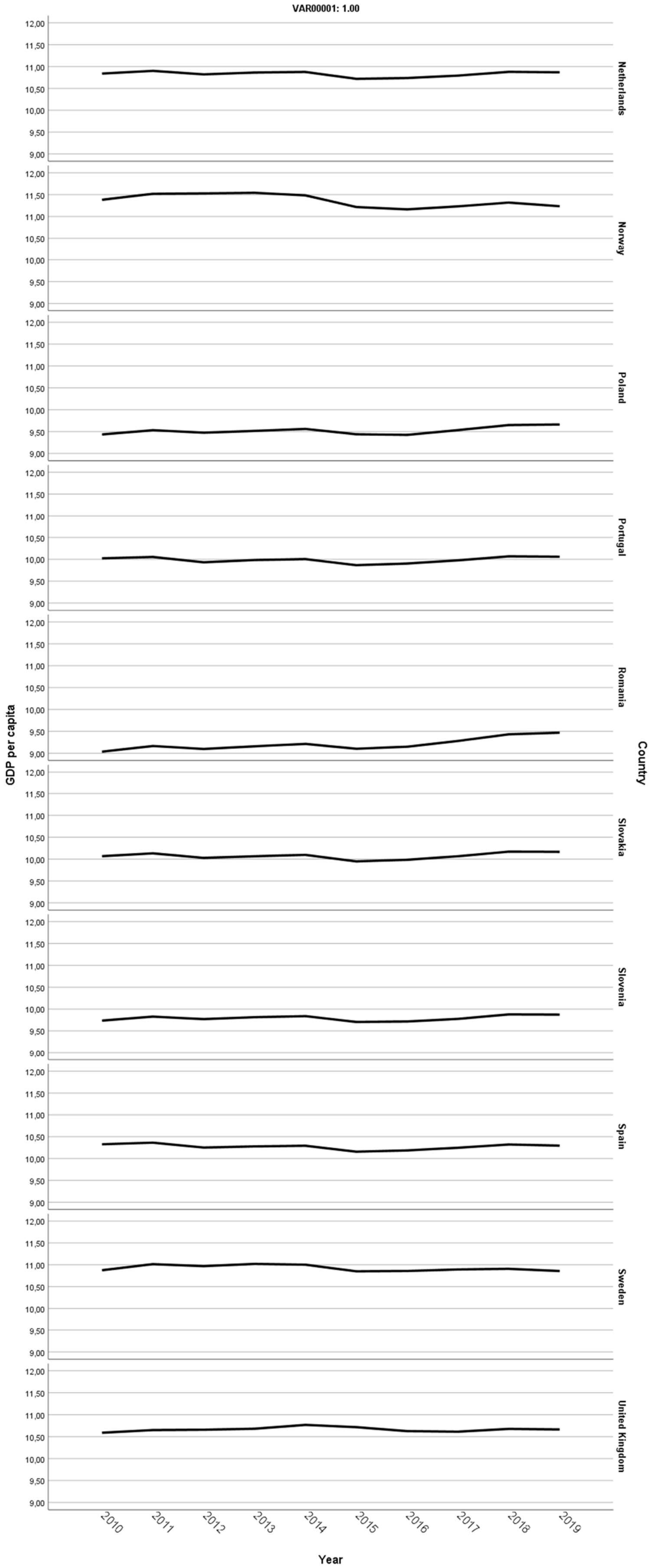

VAR00001: 1.00
GDP per capita
Country
Netherlands
Norway
Poland
Portugal
Romania
Slovakia
Slovenia
Spain
Sweden
United Kingdom
12,00
11,50
11,00
10,50
10,00
9,50
9,00
2010
2011
2012
2013
2014
2015
2016
2017
2018
2019
Year

Figure S8. Trends in the governance index between 2010-2019 across 30 European countries.

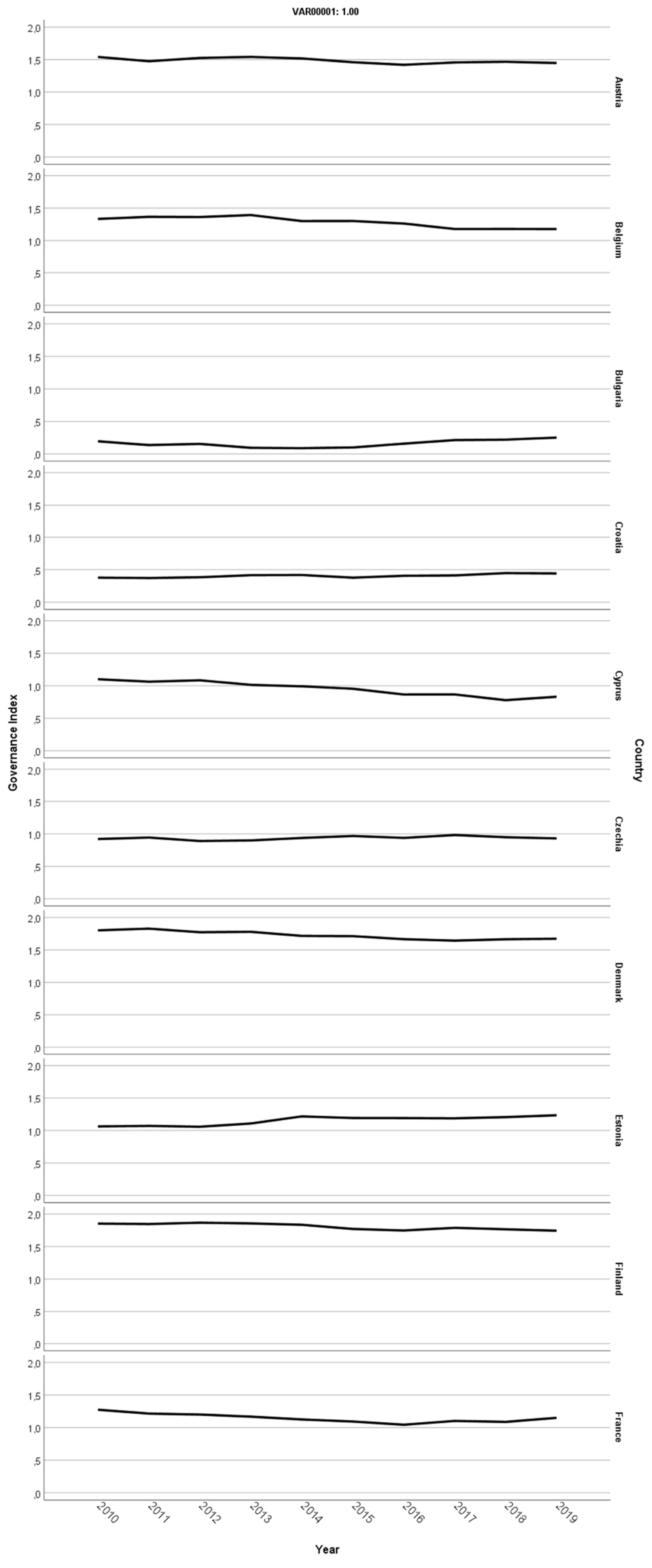

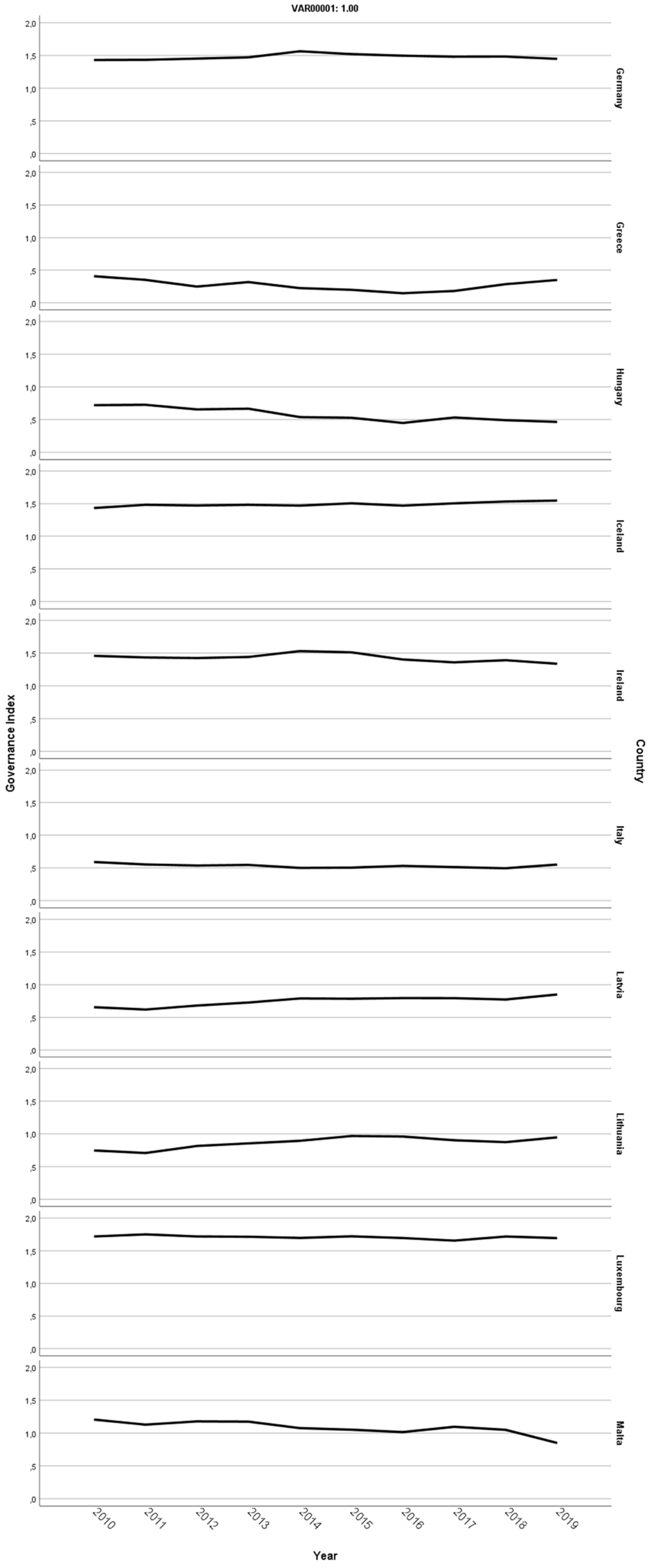
VAR00001: 1.00
Governance Index
Country
Germany
Greece
Hungary
Iceland
Ireland
Italy
Latvia
Lithuania
Luxembourg
Malta
2,0
1,5
1,0
,5
,0
2010
2011
2012
2013
2014
2015
2016
2017
2018
2019
Year

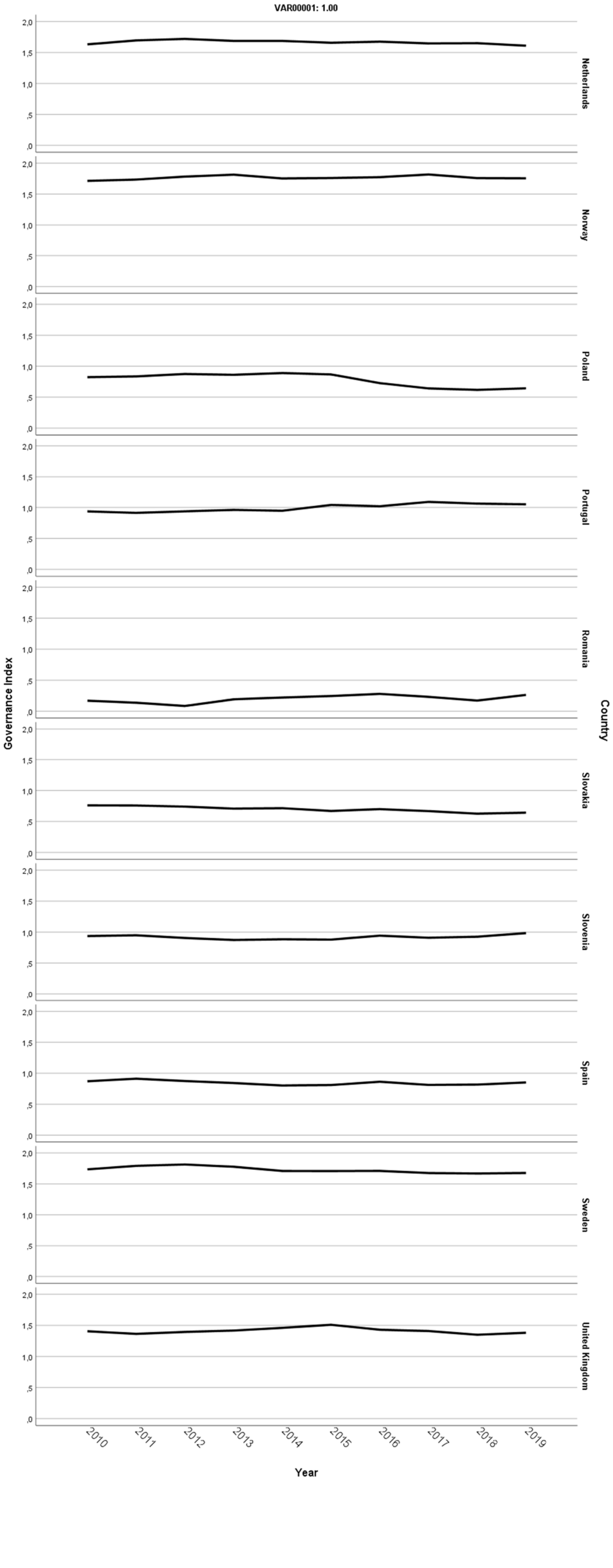

VAR00001: 1.00
Governance Index
Country
Netherlands
Norway
Poland
Portugal
Romania
Slovakia
Slovenia
Spain
Sweden
United Kingdom
2,0
1,5
1,0
,5
,0
2010
2011
2012
2013
2014
2015
2016
2017
2018
2019
Year

Figure S9. Association of antimicrobial resistance (normalized) with GDP per capita (A) and the governance index (B). Unadjusted weighted linear trend lines are shown.

A

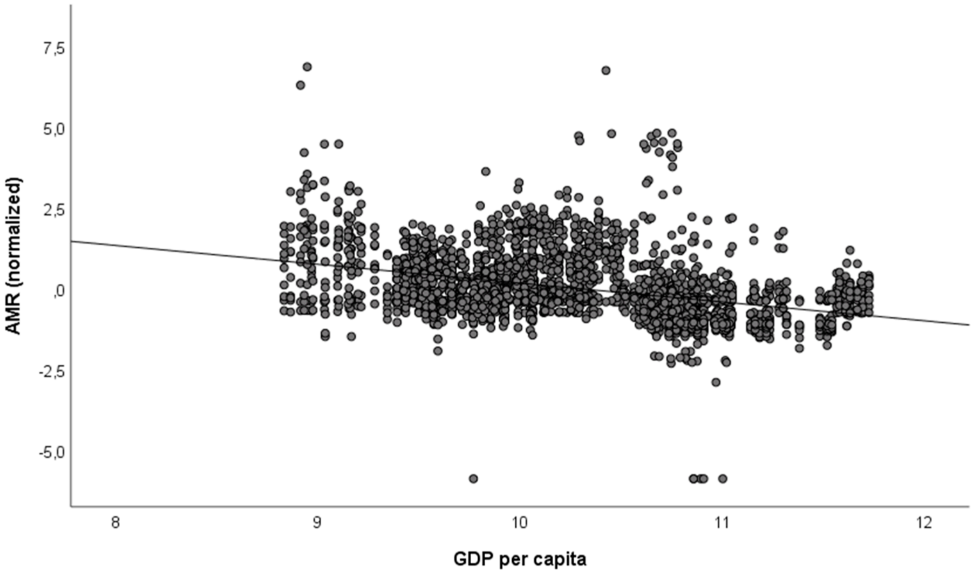

B

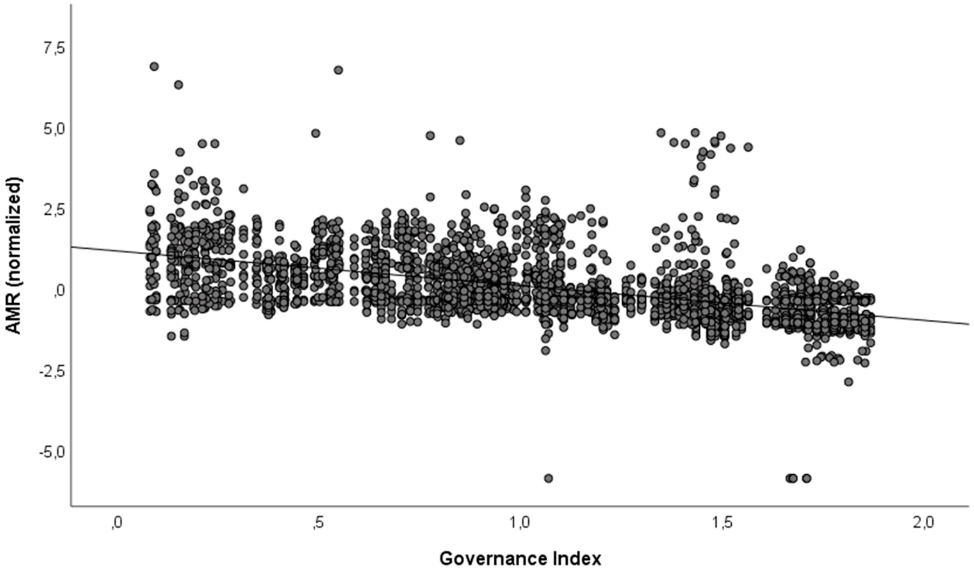

Table S1. Unadjusted and adjusted multivariable analyses to evaluate the linear relationship of antimicrobial resistance with temperature change and other predictors

Linear regression model	Coefficient (95% confidence interval)
**Unadjusted**	
Mean annual temperature change (°C)	0.042 (-0.016; 0.101)
**Adjusted Model 1[a]**	
Mean annual temperature change (°C)	0.140 (0.039; 0.241)**
Year	0.032 (-0.001; 0.065)
Interaction	-0.010 (-0.030; 0.010)
Antibiotic consumption in the community (DDD/1,000 inhabitants/day)	1.163 (1.044; 1.282)***
Population density (persons/km^2)	0.165 (0.135; 0.196)***
**Adjusted Model 2[b]**	
Mean annual temperature change (°C)	0.027 (-0.064; 0.118)
Year	0.051 (0.022; 0.081)**
Interaction	-0.017 (-0.035; 0.001)
Antibiotic consumption in the community (DDD/1,000 inhabitants/day)	0.506 (0.366; 0.646)***
Population density (persons/km^2)	0.143 (0.116; 0.170)***
GDP per capita (current US$)	0.093 (-0.022; 0.209)
Governance Index	-1.043 (-1.207; -0.879)***

Abbreviations: DDD, defined daily dose; GDP, gross domestic product.

For interpretability, year was zeroed at baseline (2010) and a natural log transform was applied to antibiotic consumption, GDP per capita, and population density to improve linear fit.

[a] Coefficients with 95% confidence intervals) were adjusted for country, mean annual temperature change, year, antibiotic consumption, population density, and the interaction between year and temperature change.

[b] Coefficients with 95% confidence intervals) were adjusted for country, mean annual temperature change, year, antibiotic consumption, population density, GDP per capita, governance index, and the interaction between year and temperature change.

*** $p < 0.001$; ** $p < 0.01$
